# Clinical Significance of Serum IL-12 Level in Patients with Early Breast Carcinoma and Its Correlation with Other Tumor Markers

**DOI:** 10.3889/oamjms.2015.106

**Published:** 2015-11-03

**Authors:** Samar Samir Youssef, Manal Moussa Mohammad, Lobna R. Ezz-El-Arab

**Affiliations:** 1 *National Research Center, Microbial Biotechnology Department, Cairo, Egypt*; 2 *National Research Center, Medical Physiology Department, Medical Research Division, Cairo 11331, Egypt*; 3 *Faculty of Medicine, Ain Shams University, Radiation Oncology and Nuclear Medicine, Cairo, Egypt*

**Keywords:** Breast cancer, hormone receptor, prognosis, tumor markers, IL-12

## Abstract

**AIM:**

To investigate the diagnostic significance of Interleukin 12 (IL-12) in breast cancer (BC) and its correlation with other tumor markers including cancer antigen 15-3 (CA 15-3), carcinoembryonic antigen (CEA), matrix metalloproteinase-9 (MMP-9), tissue inhibitor of metalloproteinases-1 (TIMP-1), and MMP9/TIMP1 ratio.

**METHODS:**

Serum levels of IL-12, tumor markers, and hormone receptors were measured in 92 BC and 56 benign lesion patients versus 40 healthy subjects. Clinical stage, tumor size, lymph node metastasis, grade, and histological type were recorded.

**RESULTS:**

BC patients have lower IL-12, but higher CA 15.3 and CEA than control group. High levels of serum IL-12 were associated with lymph node positivity and progesterone receptor negativity. IL-12 was significant lower in invasive ductal carcinoma (IDC) compared to non IDC histological type. IL-12 was higher in patients with higher stage and grade but the difference was not statistically significant. IL-12 correlates negatively with MMP9/TIMP1 ratio.

**CONCLUSION:**

IL-12 is less specific than CEA for screening early BC, but its correlation with tumor aggressiveness and progression markers may have a prognostic value.

## Introduction

Breast cancer is reported to occur at a higher rate in women of advanced countries than do other cancers [1]. The incidence and mortality from BC in developing countries are expected to increase in the near future due to the trend of living westernization. According to Ferly 2008, BC accounts for 38% of all new cancer cases in Egypt [2]. In most BC cases, the patients neither practice self examination nor have detectable symptoms. Therefore, it is very important to find markers for early diagnose to reduce sequel and mortality.

The etiology of BC is multifactorial, and its clinical course and molecular and pathological features are highly diverse [3]. Estrogen receptor (ER), progesterone receptor (PgR), and human epithelial growth factor receptor 2 (HER2) are recognized as common clinical markers for tumor growth and progression, and indicators for determining appropriate therapy for BC patients [4]. Similarly, concentrations of both Matrix metalloproteinase-9 (MMP-9) and Tissue inhibitor of metalloproteinases-1(TIMP-1) have been suggested as useful biomarkers for predicting BC progression and thus patient survival [5].

Both innate and acquired arms of the immune system are believed to play crucial roles in the anti- tumor response, and the interaction between host immune system and tumor cells has been the subject of intense research over the past decades [6, 7]. Cytokines are intercellular short-acting soluble mediators produced by immune cells and are involved in the pathogenesis of cancer. Changes in cytokine levels mediated directly or indirectly by the tumor are important parameters that affect the course of disease [3].

Interleukin-12 (IL-12) is a cytokine that plays an essential role in both innate and adaptive arms of immunity [8]. IL-12 is known to play a fundamental role in activating anti tumor immunity; it induces T-cell differentiation and activates natural killer cells [8]. It is also an antiangiogenic agent [9, 10] that enhances anti tumor activity in preclinical models [11]. Endogenous IL-12 is required for resistance to many pathogens and to transplantable and chemically induced tumors. In experimental tumor models, recombinant IL-12 treatment has a dramatic anti- tumor effect on transplantable tumors. It has been previously shown that IL-12 also regulates the levels of MMP-9 and TIMP-1 in the tumor micro-environment of BC model [12]. Nonetheless, studies investigating the correlation of IL-12 with MMP9 and TIMP1 in BC patients are lacking.

The studies that evaluated the predictive and prognostic significance of serum IL-12 levels in BC patients; in addition to its correlation with other prognostic markers are few and have conflicting results [13-16]. Moreover, studies investigating the correlation between IL-12 expression and MMP9 and TIMP1 are lacking. Therefore, the aim of this study was to evaluate the serum level of IL-12 at different stages of BC and its correlation with other prognostic tumor markers in BC patients compared to benign lesion and healthy subjects.

## Materials and Methods

### Patient characteristics

The study population included consecutive 92 women with proved early stage BC, and 56 women with benign (B) breast lesions. Control (C) group included 40 apparently healthy women with no viral infections or other clinical complications. Benign lesions were randomly selected in the same time period. Serum samples were obtained at baseline, none of the patients was pre-treated.

Breast physical examination was carried out. The tumors were graded according to the modified Nottingham SBR system [17] and categorized according to TNM staging [18]. Tumor assessment was done including histopathological grade, lymph node status, and hormone receptor (ER, PgR and HER2) status.

### IL 12 and tumor marker detection

IL-12 p 40 was measured using sandwich enzyme-linked immunosorbent assay (ELISA) kits from Boster immunoleader (Boster Biological Technology Co., Inc.). ELISA kits purchased from Abcom (Cambridge, MA, USA), were used to measure serum CEA and CA15-3 protein levels per the manufacturer’s instructions. Serum concentrations of MMP-9 and TIMP-1 were determined by using human MMP-9 and TIMP-1 ELISA kits from RayBiotech (Norcross, GA) according to the manufacturer protocol.

### Hormone receptor immunohistochemistry

Immunohistochemistry was performed on paraffin-embedded tissues sections (3-4 μm-thick) using the following primary antibodies: ER-α (clone 6F11, monoclonal; Novocastra, Newcastle-Upon-Tyne, UK), PgR (clone PgR636, monoclonal; DakoCytomation, Glostrup, Denmark) and HER2 (A0485, polyclonal; DakoCytomation). Diaminobenzidine (Dako) was used as the chromogen. ER and PgR expression was considered positive when at least 10% of invasive tumoral cells exhibited nuclear staining, regardless of intensity. For HER2, the immunohistochemical score was assigned according to the Herceptest scoring system [19].

### Statistical analysis

The mean, mean rank and median levels of expression of analyzed interleukins were compared using Kruskal-Wallis. We used Pearson correlation to test the associations between different variables. The t test and one-way ANOVA were performed to calculate the P value. The threshold value for optimal sensitivity and specificity of MMP-9, TIMP-1, MMP-9/TIMP-1 were determined by Receiver Operating Characteristics (ROC) curve, which was constructed by calculating the true-positive fraction (sensitivity %) and false-positive fraction (100- specificity %) of the above-mentioned markers at several cut-off points [12]. The P values were considered statistically significant if P < 0.05.

## Results

### Clinical characteristics of subjects

All included subjects were females with age range 17-67 years, there wasn’t a significant difference in the mean age and body mass index between BC, and benign lesion patients and control subjects (*P* = 0.143, *P* = 0.2). Among 92 BC patients, 64 had positive ER, 56 had positive PgR, 60 had positive HER2. 36 were lymph node positive. According to their clinical stage, 52 patients were stage I and 40 patients were stage II. Regarding grade, there have been 64 grade I, and 28 grade II patients. Patients were divided according to the histological type of the tumor to invasive ductal carcinoma (IDC, n = 64) and non-invasive ductal carcinoma (NIDC, n = 28).

### Serum levels of IL-12, CEA and CA

Serum CEA and CA levels were statistically significantly lower in control (C) than both benign (B) and Breast cancer (BC) subjects as shown in Table 1.

**Table 1:** IL-12 and biomarkers serum levels in BC, benign and control subjects

Groups	Median of serum values		
	CEA (ng/ml)	CA15-3 (ng/ml)	IL-12 (pg/ml)

Control (N=40)	0.8250	10.900	170

Benign (N=56)	0.900	10.450	130

BC (N=92)	16.00	72.000	53.6

Significance (*P*)	0.0001	0.0001	0.0001

N:number, *P*: *P* value

On the contrary, IL-12 serum level was significantly higher in control compared to BC and benign tumor patients. Table 2 shows that IL-12 has similar specificity but 10% less sensitivity than CEA and CA.

**Table 2:** ROC curve analysis results for comparison of diagnostic efficiency of IL-12 compared to CA15-3 and CEA

	AUC	95% CI (SE)	Sensitivity (%)	Specificity (%)
CEA	0.870	0.794 - 0.925 (0.0496)	91.3%	94.1%

CA15-3	0.873	0.797 - 0.928 (0.0491)	95.7%	97.1%

IL-12	0.664	0.569 - 0.750 (0.0588)	82.6%	94.9%

### Correlation of IL-12 and tumor markers with patients’ clinical status

We compared median levels of serum IL-12 and other tumor markers with the patients’ clinical status; the results are shown in Table 3. IL-12 was significantly higher in hormone receptor negative, LN positive, and NIDC patients. Higher stage and grade were associated with higher IL-12 level, but the difference was not statistically significant.

**Table 3:** IL-12, CEA, CA, TIMP1, MMP9 and clinicopathological markers

Parameter	N	IL-12 Median (pg/ml)	CEA Median (ng/ml)	CA15.3 Median (ng/ml)	MMP9 Median (ng/ml)	TIMP1 Median (ng/ml)	MMP9/TIMP1
ER:							
+ve	64	46.05	16.0	73.5	53.9	142.4	0.391
-ve	28	128.8	16.0	67.0	48.6	116.8	0.404

*P* value		0.08	.972	0.265	0.552	0.001*	0.700

PgR							
+ve	56	41.7	15.5	69.5	53.6	141.5	0.394
-ve	36	128.8	18.0	77.0	53. 7	121.5	0.395

*P* value		0.008*	0.328	0.95	0.644	0.024*	0.988

HER2							
+ve	60	50.59	16.0	67.0	54.46	141.3	0.397
-ve	32	100.5	18.0	76.5	50.72	127.7	0.389

*P* value		0.053*	0.364	0.836	0.62	0.055	0.84

LN							
+ve	36	133	16.0	67.0	56.25	135.7	0.352
-ve	56	36.05	16.0	73.0	47.72	136.6	0.422

*P* value		0.014*	0.828	0.129	0.011*	0.589	0.023*

Stage							
I	52	50.0	17.0	77.0	58.6	140.3	0.427
II	78.3	40	16.0	65.5	47.6	126.5	0.353

*P* value		0.879	0.416	0.021*	0.0001*	0.036*	0.013*

Grade							
Low grade	64	51.8	16.0	67.0	49.7	136.6	0.384
High grade	28	68.0	24.0	83.0	57.8	135.7	0.42

*P* value		0.251	0.132	0.131	0.098	0.652	0.27

Histological							
type							
IDC	64	46.05	14.5	66.5	55.42	137.1	0.413
NIDC	28	130.24	20.0	78.0	51.02	132.6	0.352

*P* value		0.019*	0.096	0.414	0.05	0.5	0.059

Parameter	N	IL-12	CEA	CA15.3	MMP9	TIMP1	MMP9/TIMP1
	92	Median (pg/ml)	Median (ng/ml)	Median (ng/ml)	Median (ng/ml)	Median (ng/ml)	

ER:							
+ve	64	46.05	16.0	73.5	53.9	142.4	0.391
-ve	28	128.8	16.0	67.0	48.6	116.8	0.404

*P* value		0.08	.972	0.265	0.552	0.001*	0.700

PgR							
+ve	56	41.7	15.5	69.5	53.6	141.5	0.394
-ve	36	128.8	18.0	77.0	53. 7	121.5	0.395

*P* value		0.008*	0.328	0.95	0.644	0.024*	0.988

HER2							
+ve	60	50.59	16.0	67.0	54.46	141.3	0.397
-ve	32	100.5	18.0	76.5	50.72	127.7	0.389

*P* value		0.053*	0.364	0.836	0.62	0.055	0.84

LN							
+ve	36	133	16.0	67.0	56.25	135.7	0.352
-ve	56	36.05	16.0	73.0	47.72	136.6	0.422

*P* value		0.014*	0.828	0.129	0.011*	0.589	0.023*

Stage							
I	52	50.0	17.0	77.0	58.6	140.3	0.427
II	40	78.3	16.0	65.5	47.6	126.5	0.353

*P* value		0.879	0.416	0.021*	0.0001*	0.036*	0.013*

Grade							
Low grade	64	51.8	16.0	67.0	49.7	136.6	0.384
High grade	28	68.0	24.0	83.0	57.8	135.7	0.42

*P* value		0.251	0.132	0.131	0.098	0.652	0.27

Histological							
type							
IDC	64	46.05	14.5	66.5	55.42	137.1	0.413
NIDC	28	130.24	20.0	78.0	51.02	132.6	0.352

*P* value		0.019*	0.096	0.414	0.05	0.5	0.059

* significant at p < 0.05

Early stage of BC was found to be significantly associated with higher levels of CA15.3, MMP9, TIMP1, and MMP9/TIMP1 ratio. Higher MMP9 expression was significantly associated with LN positivity. Higher TIMP1 level was associated with positive ER and PgR.

### Correlation of IL-12 with other prognostic markers in BC patients

The statistical correlation between serum levels of prognostic markers with IL-12 in BC was investigated. Results showed statistically significant correlation between serum levels of IL-12 with MMP/TIMP ratio, CEA cut 5, CEA 15.3=30 in BC (table 4).

**Table 4:** Association of IL-12 expression with prognostic markers of BC

		TIMP	MMP	MMP/TIMP	CEA cutoff=5	CA15.3=30
IL2	Pearson Correlation	0.193	-0.192	-0.352	-0.375	-0.362

	Sig. (2-tailed)	0.200	0.201	0.017*	0.010*	0.013*

	N	92	92	92	92	92

* significant at p < 0.05

## Discussion

Cytokines play varied roles in cancer pathogenesis, with increasing evidence suggesting their involvement in tumor initiation, growth and metastasis [20]. IL-12 is a proinflammatory cytokine. The potent anti tumor activity of IL-12 has been demonstrated in many preclinical murine tumor models [21-23]. Moreover, an earlier study proved that cancer patients with elevated blood concentrations of IL-12 have a higher survival rate than patients with low concentrations [24]. Previous studies investigating the clinical significance of serum levels of IL-12 in BC are few and showed conflicting results. This study is devoted to investigate the clinical significance of IL-12 expression in BC and to deduce its correlation with CA15-3, CEA, MMP9, TIMP1 as breast tumor markers.

Results of this study for assessing the role of IL-12 in early diagnosis of BC patients by ROC curve analysis showed that the highest specificity obtained for IL-12 was 82% at cut off 147pg/ml. This sensitivity is not efficient for screening for early BC. Moreover, IL-12 sensitivity was 10% less that of CEA which is the marker commonly used to screen BC, and consequently IL-12 is not effective for screening and diagnosis of early BC patients.

Derin et al. [14] and Rao et al. [15] reported no significant difference between BC patients and healthy control serum IL-12. Our results reported a significant deficiency in IL-12 expression in BC patients than benign tumor patients than healthy subjects, a result that is consistent with Merendino et al. [9]. Although our results disagree with Hussein et al. [13] and Chavey et al. [18] who reported higher levels of IL-12 in BC patients than control subjects, this conflict may be attributed to the hormone receptor (ER, PgR and HER2) status, since similar to their findings, we recorded higher levels in ER, PgR and HER2 negative rather than positive patients which was statistically significant in PgR. Several reports demonstrated direct down regulation of cytokines in different organs by ER [25-28] and PR [29-32]. The inverse correlation between IL-12 and hormone receptors status may reflect the greater aggressiveness of this subtype of breast tumors, since the use of IL-12 as anti tumor is directed to induce or increase hormone sensitivity [33].

The correlation between serum level of IL-12 and clinicopathological parameters in BC patients is scarcely discussed and results were controversial. The current study revealed that IL-12 serum levels were statistically higher in NIDC and LN positive compared to IDC and LN negative patients, respectively. This indicates that IL-12 may have noninvasive prognostic value in BC patients. This is consistent with the previous Egyptian study of Hussein et al., 2004 and also with that of Rao et al., 2007 and Chavey et al., 2007.

Many publications support the link between MM9, TIMP-1 and tumor cell survival demonstrating a high statistically significant association between high tumor or plasma levels of TIMP-1 and poor cancer patient outcome [5, 34, 35]. It was previously shown that IL-12 regulates MMP9 and TIMP1 in the tumor micro enviroment leading to reduced MMP9 and increased TIMP1 levels in IL-12 treated tumors [12]. But up to our knowledge, this is the first study investigating the correlation between serum levels of IL-12 and MMP9, TIMP1 and MMP9/TIMP1 in BC. Notably, our result recorded a negative correlation between serum level of IL-12 and MMP9/TIMP1 ratio, supporting earlier reports and pinpointing the importance of monitoring disease progression using both immune components and proteolytic markers. Similarly, this is the first time to assess, the correlation between serum levels of IL-12, CA and CEA in BC patients; the statistically significant negative correlation reported here confirms the role of IL-12 IL-12 levels in progression of disease as these markers are mainly related to disease progression.

In conclusion, IL-12 may not be the biomarker of choice for diagnosis and screening of early BC, nonetheless it should be considered as potential prognostic markers, especially in combination with other prognostic markers like MMP9, TIMP1, CEA and CA in early BC patients.
